# Contribution of Ionotropic Glutamatergic Receptors to Excitability and Attentional Signals in Macaque Frontal Eye Field

**DOI:** 10.1093/cercor/bhab007

**Published:** 2021-02-24

**Authors:** Miguel Dasilva, Christian Brandt, Marc Alwin Gieselmann, Claudia Distler, Alexander Thiele

**Affiliations:** Institute of Neuroscience, Newcastle University, Newcastle upon Tyne NE2 4HH, UK; College of Medicine and Health, University of Exeter, EX1 2LU, UK; Institute of Neuroscience, Newcastle University, Newcastle upon Tyne NE2 4HH, UK; Institute of Clinical Research, University of Southern Denmark, DK-5230 Odense, Denmark; Institute of Neuroscience, Newcastle University, Newcastle upon Tyne NE2 4HH, UK; Allgemeine Zoologie und Neurobiologie, Ruhr-Universität Bochum, Bochum 44801 Germany; Institute of Neuroscience, Newcastle University, Newcastle upon Tyne NE2 4HH, UK

**Keywords:** attention, frontal cortex, neuropharmacology, primate

## Abstract

Top-down attention, controlled by frontal cortical areas, is a key component of cognitive operations. How different neurotransmitters and neuromodulators flexibly change the cellular and network interactions with attention demands remains poorly understood. While acetylcholine and dopamine are critically involved, glutamatergic receptors have been proposed to play important roles. To understand their contribution to attentional signals, we investigated how ionotropic glutamatergic receptors in the frontal eye field (FEF) of male macaques contribute to neuronal excitability and attentional control signals in different cell types. Broad-spiking and narrow-spiking cells both required *N*-methyl-D-aspartic acid and α-amino-3-hydroxy-5-methyl-4-isoxazolepropionic acid receptor activation for normal excitability, thereby affecting ongoing or stimulus-driven activity. However, attentional control signals were not dependent on either glutamatergic receptor type in broad- or narrow-spiking cells. A further subdivision of cell types into different functional types using cluster-analysis based on spike waveforms and spiking characteristics did not change the conclusions. This can be explained by a model where local blockade of specific ionotropic receptors is compensated by cell embedding in large-scale networks. It sets the glutamatergic system apart from the cholinergic system in FEF and demonstrates that a reduction in excitability is not sufficient to induce a reduction in attentional control signals.

## Introduction

Top-down attention improves sensory processing by altering firing rates, rate variance and rate covariance in visual cortex (Moran and Desimone 1985; Spitzer et al. 1988; Treue and Maunsell 1996; Roelfsema et al. 1998; Mitchell et al. 2007; Roberts et al. 2007; Cohen and Maunsell 2009; Mitchell et al. 2009). The effect of different neuromodulators and neurotransmitters on attentional signals has been elucidated in different model systems and in different cortical areas over the past 2 decades. For example, acetylcholine and glutamate, through its action on *N*-methyl-D-aspartic acid receptors (NMDAR) are critically involved in the neuronal mechanism supporting attention in primary visual cortex (Herrero et al. 2008; Self et al. 2012; Herrero et al. 2013), and acetylcholine and dopamine are involved in attentional signaling in the frontal eye field (FEF) (Noudoost and Moore 2011; Dasilva et al. 2019). While the role of NMDARs in attention control signals in the frontal cortex has not been explicitly tested, NMDARs are critically involved in spatial working memory signals and rule-based activity in dorsolateral prefrontal cortex (DLPFC) (Skoblenick and Everling 2012; Wang et al. 2013; Skoblenick and Everling 2014; Wang and Arnsten 2015) (but see van Vugt et al. 2020). NMDARs have comparably long activation time courses, enabling neuronal circuits to generate stable mental representations, in the form of persistent firing, a feature that contributes to the generation of working memory signals (Wang 2001; Wang et al. 2013). Working memory, the ability to temporarily keep information available for processing, is conceptually similar to cued top-down spatial attention, where specific aspects of the external world need to be monitored for extended periods of time, and where the locus of attention needs to be kept activated during the process of monitoring. Thus, it is reasonable to speculate that NMDARs might also be involved in the generation of spatial top-down signals in attention controlling areas. An area heavily involved in the control of spatial top-down attention is the FEF (Corbetta et al. 2002; Moore and Armstrong 2003; Schall 2004; Wardak et al. 2006; Armstrong et al. 2009; Gregoriou et al. 2014; Bichot et al. 2015; Thiele et al. 2016). FEF neurons show strongly enhanced enduring activity for attended locations (Chang et al. 2012; Gregoriou et al. 2012; Thiele et al*.* 2016; Dasilva et al*.* 2019), and their feedback signals enhance the activity of retinotopically aligned neurons in area V4 (Moore and Armstrong 2003; Moore and Fallah 2004; Gregoriou et al. 2009; Noudoost and Moore 2011; Gregoriou et al*.* 2014). To investigate whether NMDARs contribute to attentional signals in FEF we combined pharmacological analysis of ionotropic glutamatergic receptors (IGluR: NMDA or α-amino-3-hydroxy-5-methyl-4-isoxazolepropionic acid receptor [AMPAR]) with single-cell recordings in FEF of macaque monkeys performing a feature-based spatial top-down attention task. NMDAR and AMPAR blockade resulted in reduced neuronal activity. Contrary to our prediction, IGluR blockade did not result in reduced attentional modulation, whether assessed by firing rate modulation, or by measures of neuronal variability.

## Methods

All procedures were approved by the Newcastle University Animal Welfare Ethical Review Board and carried out in accordance with the European Communities Council Directive RL 2010/63/EC, the US National Institutes of Health Guidelines for the Care and Use of Animals for Experimental Procedures, and the UK Animals Scientific Procedures Act. We used 2 adult awake male macaques (*Macaca mulatta,* age 5–9 years, weight 11–15 kg). Animals were motivated to engage in the task through fluid control at levels that do not affect animal physiology and have minimal impact on psychological well-being (Gray et al. 2016).

### Surgical Preparation

The monkeys were implanted with a headpost and recording chambers over area FEF under sterile condition and under general anesthesia. Surgery and postoperative care has been published in detail previously (Thiele et al. 2006).

### Identification of Recording Sites

Area FEF was initially identified by means of structural magnetic resonance imaging. FEF recordings sites were confirmed by visual receptive field (RF) size and topography (Bruce et al. 1985), memory-guided saccade responses (persistent activity during the memory period), by saccade-related responses to the visual/motor field (saccade field, SF), and by means of low-current (50 μA) electrical saccade induction (Bruce et al*.* 1985). The location of the recording sites in area FEF in both monkeys was verified in histological sections stained for cyto- and myeloarchitecture (Distler and Hoffmann 2001).

### RF and Saccade Field (SF) Mapping

The location and size of RF was measured by a reverse correlation method (Gieselmann and Thiele 2008). SFs were mapped as described previously (Thiele et al*.* 2016). For visually guided saccades, monkeys fixated centrally, and after 500 ms, a peripheral saccade target was presented at 1 of 9 equally spaced possible peripheral location (equidistant to the fixation spot on an invisible circle). The distance to the fixation spot depended on the estimated RF location. If no clear RF had been determined in the RF mapping procedure, we used a variety of eccentricities ranging from 5° to 15° eccentricity to determine the SF location. Monkeys had to saccade to the target location once the fixation spot was extinguished (1000 ms after saccade target onset). In a few cases we also mapped memory-guided SFs. Following fixation onset, after 500 ms the saccade target briefly flashed for 200 ms. The monkey had to memorize that location and make a saccade to the memorized location after fixation offset (800 ms after the saccade target was extinguished).

**Figure 1:**
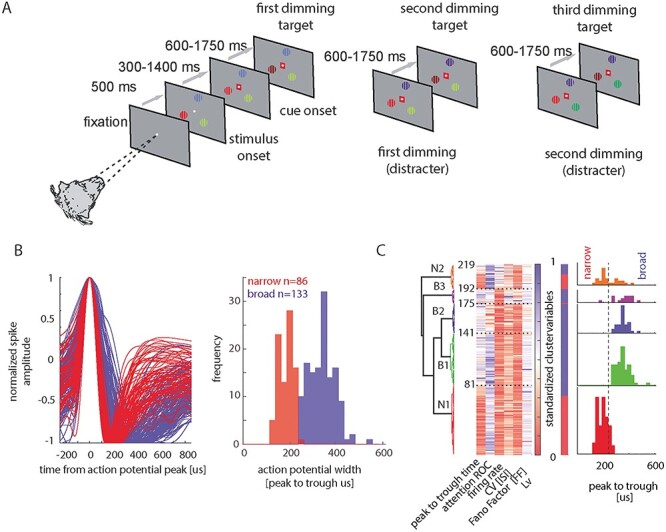
(*A*) Cartoon of the task. Monkeys fixated centrally. In total, 500 ms after fixation onset 3 colored gratings were presented equidistant from the fixation spot. One of the gratings was placed in the RF of the neuron under study. After a variable time (300–1400 ms), a central colored cue indicated which stimulus was behaviorally relevant on the current trial. The animal had to covertly monitor this stimulus and wait for it to change luminance (referred to as target dimming in the figure). The target dimming could occur first, second, or third in the sequence of dimming events (left to right in the figure). Distracter dimming had to be ignored by the monkey. Detection of target dimming was indicated by releasing a handheld touch bar. (For additional details see Methods). (*B*) Normalized spike average waveforms of all narrow-spiking (red) and all broad-spiking (blue) cells recorded and distribution of P2T times of recorded spike waveforms. Narrow-spiking P2T distribution is shown in red, broad-spiking P2T distribution is shown in blue. (*C*) Subdivision of different cell types into 5 different cell clusters. Dendrogram on the left shows the linkage between different cell clusters. Heat maps (next to the dendrograms) show the values of different neuronal features used for classification (*x*-axis) for all clustered cells (*y*-axis). Color coding is according to increasing standardized feature values (cluster variables). Dashed lines within heat maps show cell class borders along with cumulative cell numbers (i.e., cluster sizes can be inferred from these numbers). The color bar to the right of the standardized cluster-variable bar shows encoding of cell type along the narrow–broad-spiking divide (narrow: red, broad: blue). Distribution of P2T times for each cell cluster is shown on the right of each subplot. Red dashed line shows broad–narrow divide used in the current paper (240 μs).

### Behavioral Task and Stimuli

Monkeys fixated a white fixation point on a gray background presented centrally on a 20″ analogue cathode ray tube monitor (110 Hz, 1600 × 1200 pixels, 57 cm from the animal). Eye position was monitored with an infrared based system (Thomas Recording, 220 Hz) with a fixation window of ±0.7–2°. The monkey initiated trials by holding a touch bar and fixating on the central point (Fig. 1). After 500 ms three stimuli appeared on the screen, equidistant from the fixation spot. One stimulus was centered on the RF/SF of the recorded neuron. The other stimuli were presented equidistant on an invisible circle centered on the fixation spot. Stimuli were square wave gratings (1 cycle/deg duty cycle) in a circular aperture sized according to the RF size. One grating was red/gray, 1 green/gray, and 1 blue/gray. Locations of differently colored gratings were fixed within a recording session but differed pseudorandomly between recording sessions. Grating orientation was fixed within a session but differed randomly between sessions. Gratings moved perpendicular to the orientation (1 Hz temporal frequency). The motion direction (perpendicular to the orientation) was pseudorandomly assigned on each trial. After a randomly selected time of 300–1400 ms, a central cue of either green, blue, or red color appeared. The cue color indicated which of the 3 gratings would be behaviorally relevant on the current trial (the color-matched grating). Cue selection occurred pseudorandomly. After 600–1750 ms, 1 pseudorandomly selected grating was reduced in luminance (for details see Thiele et al*.* 2016). If the cued grating had changed luminance, the monkey had to release a central touch bar within 600 ms to obtain a fluid reward. If an un-cued grating had changed luminance, the animal had to ignore it and wait for the cued grating to change luminance. This could happen 600–1750 ms after the first dimming or 600–1750 ms after the second dimming (Fig. 1*A*). Throughout the entire period, the monkey had to fixate on the central fixation spot. The task had no catch trials, that is, the cued grating always changed luminance, but the order thereof was unpredictable up to the point when the second grating had changed luminance. The timing of the dimming was also unpredictable, within the time period indicated above.

### Electrophysiological Recordings and Drug Application

In this study we used the NMDAR blocker (2*R*)-amino-5-phosphonovaleric acid (APV) or the AMPA/Kainate receptor (AMPAR) blocker 6-cyano-7-nitroquinoxaline-2,3-dione (CNQX) to affect neuronal activity. Drugs were applied iontophoretically using a tungsten-in-glass electrode flanked by 2 pipettes (Thiele et al*.* 2006). The tungsten in glass electrode had impedances of 0.5–1.5 MΩ (measured at 1 kHz) and an exposed tungsten tip of <10 μm. Pipette opening diameter varied between 1 and 4 μm. Pipette resistance varied between 10 and 400 MΩ (median: 58 MΩ, 25–75%ile: 40, 90 MΩ, range: 10–400 MΩ). The integrity of the electrode and the pipettes was checked under the microscope before and after each recording sessions, in addition to measurements of the pipette impedance made before and after the recording at each recording site. The details regarding drug concentration, pH, and application current were: Hold currents for APV (0.04 M, pH 8.0) were usually +10 nA (median; 25–75%ile: +6 to +15 nA; minimum: +2 nA; maximum: +40 nA), ejection currents were usually −40 nA (median; 25–75%ile: −20 to −50 nA; minimum: −10 nA; maximum: −80 nA). Hold currents for CNQX (0.02 M, pH 8.0) were +10 nA (median, 25–75%ile:+8, +18 nA; minimum: +4 nA; maximum: +40 nA), ejection currents were usually −40 nA (median: −40 nA; 25–75%ile: −20 to −60 nA; minimum: −10 nA; maximum: −120 nA).

Drug application was continuous during blocks of “drug applied” conditions. Each block lasted for at least 36 trials (2 repetitions of: 3 attention locations ^*^ 2 directions of motion ^*^ 3 dimming times = 36), with error trials repeated at random times within a block, such that block length depended to some extent on monkey performance. On average, drug/no drug application for each block was approximately 3–9 min. For the data analysis, we removed the first 6 trials within a block from the data set, as drug effects and recovery usually occur with a slight delay of approximately 0.5 min.

We focused our attention analysis on the first predimming period. For each cell, we thus had 3 factors that we analyzed, namely drug/no drug (2 levels), attention location (3 levels), and stimulus motion direction (2 levels).

We did not perform many control experiments, as we have performed these types of controls on many occasions under virtually identical conditions many times before and never encountered an effect of pH or current level on neuronal activity (Roberts et al. 2005; Zinke et al. 2006; Herrero et al*.* 2008; Thiele et al. 2012; Herrero et al*.* 2013; Herrero et al. 2017). In line with this in our control experiments here (*n* = 3), we used saline in the pipettes (0.9%, pH 8.0), using similar hold and ejection currents (+20 nA hold, −40 nA eject). We did not see significant effects of saline application on firing rate or attentional modulation in any of the 3 experiments (*n* = 5 neurons).

Neurons were further analyzed if at least 10 trials per condition were available. The median number of trials for our APV recordings were *n* = 60 per condition (25, 75%ile: *n* = 53, *n* = 72). For the CNQX recordings, the median number of trials was *n* = 62 per condition (25, 75%ile: *n* = 50, *n* = 73).

### Data Collection

Stimulus presentation and behavioral control was managed by Remote Cortex 5.95 (Laboratory of Neuropsychology, National Institute for Mental Health, Bethesda, MD, http://dally.nimh.nih.gov/). Neuronal data were collected by Cheetah data acquisition (Neuralynx) interlinked with Remote Cortex. Raw data were acquired at a sampling frequency of 32 556 Hz with a 24-bit analog-to-digital converter, with minimum and maximum input ranges of 11 and 136 986 μV, respectively (preset by Neuralynx, Inc.), a direct memory access (DMA) buffer count of 128, and a DMA buffer size of 10 ms, using a 64-channel Digital Lynx 16SX Data Acquisition System (Neuralynx, Inc.). Digital referencing of voltage signals was performed prior to the recording of raw data, using commercially provided Cheetah 5 Data Acquisition Software v. 5.4.0 (Neuralynx, Inc.).

Following each recording session, the raw data were processed offline using both commercial (Neuralynx, Inc.) and custom-written (Matlab, Mathworks) software. Signals were extracted using Cheetah 5 Data Acquisition Software. The sampling frequency remained the same (32 556 Hz), whereas the input range settings were individually tailored to session, with band-pass filter frequency set to a low-cut frequency of 600 Hz and a high-cut frequency of 9000 Hz and saved at 16-bit resolution. Following extraction of thresholded spike waveforms, single-unit action potentials were extracted manually using Neuralynx Spikesort 3D v2.5 software.

### Data Analysis

We only analyzed neuronal activity associated with correct trials in the context of this paper. Data analysis was performed using custom written scripts in Matlab (Mathworks, various versions ranging from Matlab 2014–2019), We aligned neuronal activity to the stimulus, to the cue and to the first dimming onset. To analyze the effects of attention on neuronal firing rate, we quantitatively analyzed the activity from −500 ms to 0 ms before the first dimming occurred (see below for additional analysis periods to quantify different aspects). Given that there were 3 attention conditions (attend RF and 2 attend away conditions), 2 different stimulus motion directions, and 2 drug conditions (applied vs. not applied), we had 6 conditions total for each drug condition. We calculated a 3-factor analysis of variance (ANOVA) for the predimming activity to determine whether attention, drug application, and direction of motion had a significant effect on neuronal activity and whether there was a significant interaction between any of these factors. Cells that showed a significant main effect of attention during the period before the first dimming or a significant interaction (*P* < 0.05) were classified as attention modulated; cells that showed a significant main effect of drug application during the period preceding the first dimming or an interaction of drug applications with any of the other factors were classified as drug modulated. Table 1 gives an overview over the cells recorded for each of the 2 monkeys, under a given drug regime, and the number of cells that were modulated by attention, by drug application, and byboth.

**Table 1:** Number of cells recorded from the 2 monkeys under the different drug regimes, and number of cells that showed significant attention effects, number of cells with significant drug effects, and number of cells that showed significant attention and significant drug effects

	Cell type (spike) waveform	Drug type	Total *n*	Attention effect (*n*)	Drug effect (*n*)	Attention and drug effect (*n*)
Monkey 1	Narrow	APV	23	22	16	16
	Broad	APV	26	24	16	16
	Narrow	CNQX	22	17	13	12
	Broad	CNQX	12	10	9	7
Monkey 2	Narrow	APV	30	29	21	20
	Broad	APV	56	50	38	33
	Narrow	CNQX	11	11	6	6
	Broad	CNQX	39	32	24	20
Both monkeys	Narrow	APV	53	51	37	36
	Broad	APV	82	74	54	49
	Narrow	CNQX	33	28	19	18
	Broad	CNQX	51	42	33	27

### Quantification of Attentional Modulation

To investigate effects of attention on neuronal firing rates we calculated all activity levels in absolute terms and also relative to precue activity, as this was the level of activity present in the absence of directed attention. The strength of attentional modulation was quantified using an ideal observer-based approach, whereby we calculated the area under the receiver operating characteristic curve (AUROC), separately for the no drug and for the drug conditions. It is based on signal detection theory that calculates the overall probability that a random sample of neuronal activity (i.e., spikes/second) selected during one attention condition is larger than a sample selected in the alternative attention condition (Green and Swets 1966; Tolhurst et al. 1983; Thiele et al. 1999; Thiele et al. 2000, 2001).

### Quantification of Drug Effects

Drug effects were assessed by calculating a drug modulation index (MI) for each stimulus condition (*n* = 6, see above), and for each analysis period (100–400 ms after stimulus onset, 100–400 ms after cue onset, −500 to 0 before the first dimming) using the following formula:

$$ DrugMI=\frac{Activity\ no\ drug- Activity\ drug}{Activity\ no\ drug+ Activity\ drug}$$

yielding 6 different DrugMIs for each analysis period. The results for attend RF and attend away DrugMIs were qualitatively similar when analyzed separately, and we thus report a DrugMI value that was averaged across attend RF and attend away conditions.

### Quantification of Drug Effects on Attentional Modulation

The effects of drug application on attentional modulation were assessed by calculating AUROC based on the activity distributions associated with attend RF versus attend away condition (Britten et al. 1992; Britten et al. 1996). This yields 2 AUROC values for each cell recorded, one for the control condition, the other for the drug applied condition. In addition we calculated Cohen’s *D*′ as:

$$ {D}^{\prime }=\frac{{mean\ rate}_{attend\ RF}-{mean\ rate}_{attend\ away}}{pooled\ standard\ deviation\ of\ the\ rates}$$

The effects of drug application for the different cell types at the population level were quantified by a *t*-test.

### Statistic Reporting

All statistics were based on 2-sided tests and are reported as *t*-statistics, *F*-statistics for parametric tests, and as *z*-statistics for nonparametric tests. For all tests, effect size are provided along with confidence intervals (CI), where appropriate. Effects sizes are reported as Cohen’s *D* for independent measures, Cohen’s *D*_z_ for repeated measures, and as explained variance (η^2^_p_) for multifactor analyses, according to (Lakens 2013).

### Drug Levels Applied and Their Potential Implication on Activity Changes

Drug application currents varied between experiments. The reason we varied the currents is that in each experiment we aimed to modulate the neuronal activity slightly, without altering it too much. Based on our previous measurements in area V1 (Herrero et al*.* 2013), we started with low application currents (~ − 10 nA) in a few experiments. However, during these exploratory experiments drug effects with either CNQX or APV were usually limited, if present at all. We therefore ended up using larger application currents (mean APV: −39.3 nA; mean CNQX: −43.2 nA). A first concern is whether drug application currents differed systematically between APV and CNQX experiments. This was not the case. The average application current for APV experiments was −37.0 ± 23.1 nA, whereas it was −41.8 ± 22.6 nA when CNQX was applied (*t*_2,217_ = 1.2, *P* = 0.198, CI: −2.08, 10.01, Cohen’s *D*: 0.174, 2-sided *t*-test).

A second, more important, concern is whether drug applications differed for cells affected versus not affected by a drug. In any given experiment, we monitored the effect of drug application for a few trials, before settling on a level that was then used for the remainder of the day. No significant difference in drug application current was found for affected versus nonaffected cells when APV was applied (affected cells: −38.4 ± 17.0 nA, nonaffected cells: −41.4 ± 20.4 nA, *t*_1,133_ = 0.9, *P* = 0.369, CI: −3.59, 9.62, Cohen’s *D*: 0.165, 2-sided *t*-test). For the sample tested with CNQX, drug application levels equally did not differ between affected and nonaffected cells (affected cells: −43.8 ± 27.3 nA, nonaffected cells: −42.6 ± 27.5 nA, *t*_1,82_ = −0.2, *P* = 0.846, CI: −13.36, 10.98, Cohen’s *D*: 0.179, 2-sided *t*-test). Thus, different drug application levels do not account for whether a cell was affected by drug application ornot.

### Analysis of Spiking Waveforms (Broad- vs. Narrow-spiking Cells)

To classify cells as broad or narrow spiking, we performed spline interpolation of the original waveforms to obtain a resolution of 5.4 μs (Mitchell et al*.* 2007). We used peak-to-trough (P2T) time as a classification criterion. In our sample a cutoff of 240-μs P2T (Fig. 1*B*) was appropriate, as this cutoff separated the significantly bimodal distribution (calibrated Hartigan’s dip test *P* < 0.01) of P2Ts, whereas at the same time reducing the risk of classifying narrow-spiking cells as broad, since the cutoff was located to the narrow-spiking side of the bimodal separation (for details see Thiele et al*.* 2016).

### Subdividing Broad And Narrow Cell Types

Spike waveforms are widely used to subdivide cells into broad- and narrow-spiking cells, and it has regularly been assumed that these map onto putative excitatory pyramidal cells and putative parvalbumin positive fast spiking inhibitory cells, respectively. However, this mapping has been questioned repeatedly for primates (Vigneswaran et al. 2011; Soares et al. 2017). This aside, the spike waveform is still a useful grouping criterion. However, a more fine grained and detailed cell classification may require the incorporation of physiological characteristics and associated cluster analysis (e.g., Ardid et al. 2015; Dasilva et al*.* 2019). We applied the approach published by Ardid et al. (2015), for cluster analysis as outlined below.

### Cluster Analysis for the Identification of Cell Classes in FEF

#### Identifying Redundant and Uninformative Measures

For cluster identification, we prescreened the following parameters: P2T time, coefficient of variation (CV) of the interspike interval (ISI) (Holt et al. 1996; Shinomoto et al. 2003), CV of neighboring ISIs (CV2, Holt et al*.* 1996; Shinomoto et al*.* 2003), local variation (Lv) of the ISI (Holt et al*.* 1996; Shinomoto et al*.* 2003), firing rate (FR), variability of firing rate (Fano Factor [FF]), and strength of attentional modulation (AUROC). Following dissimilarity analysis, we included parameters that together explained at least 90% of the variance (Ardid et al*.* 2015). This left P2T, FR, FF, CV, Lv, and AUROC as clustering parameters.

K-means clustering was performed on standardized feature values, that is, all values were normalized to range from 0 to 1 (Dasilva et al*.* 2019).

To identify the most appropriate number of clusters K-means clustering was done using *n* = 500 realizations (with 50 replicas for each *k* and *n*, selecting the best replicate) for cluster numbers *k* = 3 to *k* = 8. Probability thresholding was performed as described in Ardid et al*.* 2015.

Final clustering was done with 50 repeats and 500 replicates for each clustering approach. Akaike information criterion (AIC) and Bayesian information criterion (BIC) informed about the most parsimonious model to be used (Burnham and Anderson 2004), whereby 5 clusters would be appropriate according to both information criteria (AIC values k = 3–8: −5519.7 −5610.3 −**5802.4** −5643.3 −5398.5 −5006.4; BIC values k = 3–8: −5397.7 −5448.3 −**5599.0** −5363.0 −5123.1 −4740.1). Numbers in bold indicate the most appropriate cluster size. 

The results for 5 clusters are shown in Figure 1*C*. The approach yielded 2 cell clusters that were exclusively comprised of broad-spiking cells (B1, B2) and one cluster that was mostly comprised of broad-spiking cells (B3). Moreover, it yielded 2 cell clusters predominantly comprised of narrow-spiking cells (N1, N2, see distribution of waveform width on the right of Figure 1*C*).

### Cell Type Separation Along Response Characteristics

**Figure 2:**
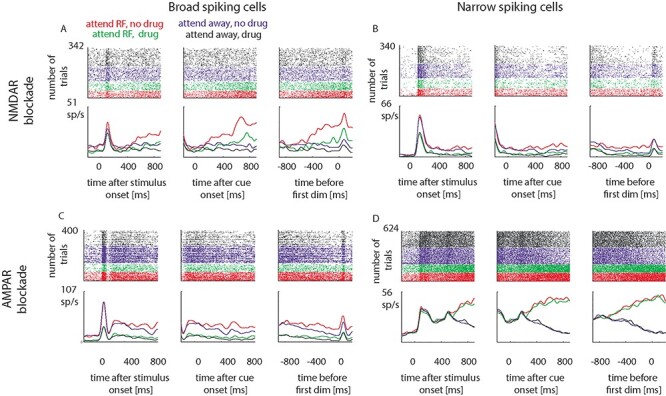
Single-cell examples of glutamatergic modulation of firing rates and of attentional effects for broad (A,C) and narrow-spiking cells (B,D), when NMDA receptors (A,B) and when AMPA receptor (C,D) were blocked. Shown are raster plots and peristimulus time histograms for the 3 main task periods (stimulus onset, cue onset, time of the first dimming), separately for the 4 attention conditions (color coded). Attention to the RF, no drug condition: red; attention away from the RF, no drug condition: blue; attention to the RF, drug condition: green; attention away from the RF, drug condition: black.

We also separated cell types along response characteristics. Traditionally, this is done by separating FEF cells into visual, visuo-movement, and movement cells, or according to visual, working memory delay activity, and saccade-related activity. While we did, at the beginning of a recording session, perform the visual- (and in some instances memory) guided saccade paradigms, cell isolation during the attention paradigm did not always match the cell isolation present during the former. It is thus difficult to assign a classification according to visual, visuomotor, and motor response types, using the SF mapping. We thus separated cells according to whether they showed mostly 1) visual-related activity (with or without some attentional modulation, an example would be in Figure 2*B*); cells that showed 2) visual activity and pronounced elevated activity toward the time of the maximal attentional modulation on attend RF trials, or 3) no or very little visual stimulus-induced activity, but strongly elevated activity before the time of the first dimming on attend RF trials. Visual cells were defined as cells that showed at least a 20% change in activity upon stimulus onset, and cells where the sustained (attend RF) activity prior to the time of the first dimming was at least 20% less than the visual-induced activity. Visual-attention cells were defined as cells that showed at least a 20% change in activity upon stimulus onset, that showed at least a 20% increased attend RF response, relative to baseline activity, and that showed the same or larger attend RF activity prior to predimming, when compared with the stimulus (transient) activity. Attention cells were defined as cells where the stimulus-induced activity was absent or small (<20% change from baseline), where the attend RF predimming response was at least 20% larger than baseline and stimulus-induced activity. Cells that did not fall into any of these classes were classified as “other response types.”

### Gain Variance Analysis

We recently demonstrated that attention-induced changes in response reliability (in FEF) are more adequately analyzed using a gain variance analysis, than using the traditionally used FF (Thiele et al*.* 2016). To investigate to what extent the different drugs affected attention- induced changes in response reliability, we thus used both FF and gain variance analysis (Goris et al. 2014). In gain variance analysis, the single trial rate (count data) is fit with a negative binomial, which yields a gain variance term. This captures the magnitude of the change in excitability from trial to trial. We used the 2 attend RF conditions to obtain an estimate of the attend RF gain variance and the 4 attend away conditions to obtain an attend away gain variance estimate. Gain variance terms were then averaged for the 2 attend RF conditions and separately for the 4 attend away conditions. This was done separately for the drug applied and the drug not applied conditions.

### Analysis of Behavioral Data

We calculated control condition and drug condition RTs and error rates for each experimental session where a significant effect of drug application was found at the cellular level. This selection was performed, as the method of drug application does not guarantee that drugs are adequately infused in every experimental session. The selection ensures that only sessions are included, where we have an independent verification that the drugs affected the neuronal tissue, a prerequisite to cause changes at the behavioral level, without preempting that a behavioral effect did occur, or its direction.

RTs were normalized relative to the session mean, whereby each session mean was calculated across all attention and all drug conditions. This normalization was done to account for differences in RF locations and sizes, which affect eccentricity and stimulus size. The latter in turn affect task difficulty and thus RTs. Effects of attention and of drug application were assessed based on the normalized single trial RTs using a 2-factor ANOVA. Post hoc testing was done based on rank-sum tests.

Error numbers (false alarms, misses) were calculated separately for the different attention conditions and for the different drug conditions. All fixation errors were discarded. These numbers were compared with the number of correct trials for the relevant condition. Effects of drug application on error rates were assessed using χ^2^ tests.

Fixational eye movements (microsaccades) were calculated from *x*- and *y*-eye movement traces that were stored with a sampling rate of 250 Hz each. Single-trial eye movement traces were extracted from 300 ms after fixation onset until the time of the first dimming and analyzed according to Engbert and Kliegl 2003, using publicly available scripts (https://github.com/alexander-pastukhov/edfImport/blob/master/edfExtractMicro-saccades.m). Single-trial microsaccade rate (Hz) were calculated from the number of microsaccades detected and the duration of the eye trace on that trial. Mean *x* and *y*-eye position after cue onset until the time of the first dimming were calculated for each trial. Single-trial data were stored along with information about the attentional condition and the drug condition and were analyzed across all recordings for a given drug (APV or CNQX).

### Network Model

We used a 2-stage neural network to determine to what extent attentional modulation can survive in a small fraction of neurons, when their NMDARs are (partly) blocked. The network model is based on the model published by Wimmer et al. 2015 (https://senselab.med.yale.edu/ModelDB/showmodel.cshtml?model=168867#tabs-1, publication). Our model (https://github.com/alex2thiele/attention-decision-model/tree/main) was adjusted in terms of network size, in terms of input (stimulus) variation over time, input current, and the balance of NMDA versus AMPA currents in the integration circuit. These changes were done to slightly reduce its winner take all characteristics and also reduce its susceptibility to even minor NMDA current drive (neurons in the original model almost completely stop firing when NMDA currents in the integration circuits are reduced by as little as 10%, due to the strong inhibition). While the original model is couched within the context of perceptual decision-making when confronted with noisy time-varying visual evidence (akin to that used in e.g., Gold and Shadlen 2000), it could equally be couched within the context of 2 populations encoding attended locations versus unattended locations. The 2 sensory populations would then represent the response to 2 identical stimuli presented at different locations (RFs). The remaining fluctuations in the sensory drive could be interpreted as small random fluctuations arising within earlier visual processing stages. A small fraction of neurons (3^*^10) in each decision population of the integration stage was selected to have reduced NMDA current drive, akin to the local iontophoresis we performed. The reduction was 40%, 25%, and 10%, respectively. Attentional bias was modeled by altering the integration-to-sensory stage feedback strength for decision population D2 (see python code for details at github site). We assessed the effect of NMDAR blockade on attentional modulation using AUROC analysis based on the stimulus induced rate of pairs of 10 neurons with equivalent NMDAR drive, when one was part of the “attend RF” population, whereas the other was part of the “attend away” population. This was repeated 100 times. We also assessed the effect of NMDAR blockade by calculating rate modulation indices for the “attend RF” population, using the population with unaffected NMDA current drive as the benchmark against the 3 NMDA current drive reduced populations (2^*^10 cells per comparison, repeated 100 times).

## Results

We recorded from 219 FEF cells from 2 monkeys (83 from M1, 136 from M2), while they performed a covert top-down spatial attention task under control conditions and with either APV (an NMDAR blocker) or CNQX (a competitive AMPAR blocker) iontophoretically applied, as described in Methods.

Monkeys performed the task as intended, reliably reporting cued dimming locations (hits), and ignoring distractor dimmings (correct rejections). Overall the mean hit rate was p(hit) = 0.998, that is, animals missed only about 1/500 target dimmings (chance-level hit rate = 0.33 [missed target dimmings 1/3]). Correct rejection rates (unreported distractor dimmings) were equally 0.998, that is, only 1/500 distractor dimmings were reported, that is, false alarm rate was (0.002). This corresponds to d-prime values of >5.67. Thus, the animals performed the task extremely well, heeding the cue and ignoring irrelevant dimmings.

For each cell, we determined whether neuronal activity was affected by the attention conditions (factor 1) or the drug application (factor 2), or whether there was an interaction between the factors, using a 2-factor ANOVA. The time period analyzed was from −500 to 0 ms before the time of the first luminance change, as this is the time period where attentional modulation in this task is most profound (Thiele et al*.* 2016). Of the 219 cells, 133 cells were classified as broad-spiking (cells with an action potential P2T time of >240 μs), whereas 86 were classified as narrow spiking (cells with an action potential P2T time of ≤240 μs). Figure 1*B* shows the spike waveforms recorded from our sample of cells that were included in the analysis (*n* = 219), and Figure 1*B* shows the distribution of the spike waveform width (P2T duration). An overview of the number of cells recorded under the different drug regimes, and the number of cells significantly affected by attention and/or drug is given in Table 1.

### Single-Cell Examples of Glutamatergic Modulation of Firing Rates and Attentional Effects

Examples of attention and drug effects on neuronal activity are shown in Figure 2. These show cells where drug applications resulted in reductions of neuronal activity and in reductions of attentional modulation. While reduction of overall neuronal activity following drug application was generally found across the population of cells, a reduction of attentional modulation was the exception and was not found consistently at the population level when using AUROC or Cohen’s *D*′ metrics assessing attentional modulation (see below). We quantified attentional modulation by calculating the AUROC, which indicates how well an ideal observer can decode the locus of attention from single-trial firing rates and by calculating Cohen’s *D*.

#### Effect of NMDAR Blockade (APV Applied) on Example Cells

Figure 2*A* shows raster plots and peristimulus time histograms of an example broad-spiking cell (P2T = 383 μs) under control conditions and when NMDARs were blocked by APV application. Attending to the RF resulted in a significantly higher firing rate than attending away (*F*_1,338_ = 228.7, *P* < 0.001, η^2^_p_ = 0.321). NMDAR blockade reduced overall activity (*F*_1,338_ = 151.28, *P* < 0.001, η^2^_p_ = 0.212). NMDAR blockade also reduced the attentional modulation in this cell (significant attention^*^drug interaction: *F*_1,338_ = 37.85, *P* < 0.001, η^2^_p_ = 0.053, AUROC_no drug_ = 0.93, AUROC_drug_ = 0.81). Figure 2*B* shows the effects of NMDAR blockade on a narrow-spiking cell (P2T = 172 μs). Attention to the RF resulted in higher firing rates than attend away trials (*F*_1,336_ = 68.7, *P* < 0.001, η^2^_p_ = 0.117), drug application reduced the firing rates (*F*_1,336_ = 189.58, *P* < 0.001, η^2^_p_ = 0.323), and it also reduced attentional modulation (attention^*^drug interaction; *F*_1,336_ = 12.79, *P* < 0.001, η^2^_p_ = 0.022, but it only mildly affected AUROC_no drug_ = 0.73, AUROC_drug_ = 0.72).

#### Effect of AMPAR Blockade on Example Cells

Figure 2*C* shows the effect of attention and AMPAR blockade on a broad-spiking cell (P2T = 382 μs). Attention to the RF increased firing rates (*F*_1,396_ = 48.75, *P* < 0.001, η^2^_p_ = 0.069). AMPAR blockade reduced overall activity (*F*_1,396_ = 241.92, *P* < 0.001, η^2^_p_ = 0.334), and it reduced attentional modulation (attention^*^drug interaction *F*_1,396_ = 7.69, *P* = 0.006, η^2^_p_ = 0.011, AUROC_no drug_ = 0.73, AUROC_drug_ = 0.63). An example for a narrow-spiking cell (P2T = 199 μs) is shown in Figure 2*D*. Attention to the RF increased firing rates (*F*_1,620_ = 792.8, *P* < 0.001, η^2^_p_ = 0.558). AMPAR blockade on its own did not affect overall activity (*F*_1,620_ = 0.16, *P* = 0.693, η^2^_p_ < 0.001), but it slightly and significantly reduced attentional modulation (attention^*^drug interaction *F*_1,620_ = 4.07, *P* = 0.04, η^2^_p_ = 0.004, AUROC_no drug_ = 0.95, AUROC_drug_ = 0.94).

### Drug Effects on General Neuronal Activity

**Figure 3:**
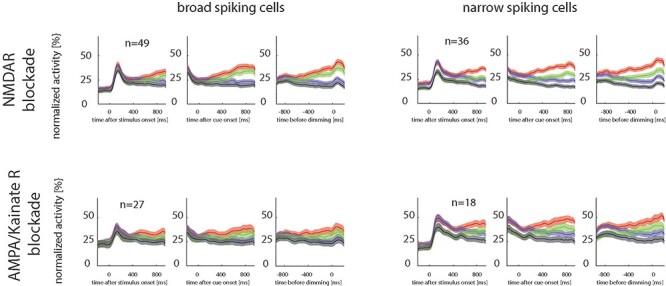
Effect of attention and of drug application on population neuronal firing rates for broad- and narrow-spiking cells, when NMDA receptors and when AMPA receptors were blocked. Lines show mean, shaded area shows SEM. Red colors: attend RF, no drug applied; green colors: attend RF, drug applied; blue colors: attend away, no drug applied; black colors: attend away, drug applied.

We will first describe the effects of glutamatergic modulation on overall neuronal gain control/excitability (which manifest as firing rate changes), followed by an analysis of how NMDAR and AMPAR antagonists affect attentional modulation. The summary effects of drug application for broad- and narrow-spiking cells are shown by the normalized population rate histograms in Figure 3.

Normalization was done for each cell by dividing the average single-cell histogram by the peak single-cell activity (derived from the respective single-cell histograms). Normalized single-cell activities were then averaged. Included are cells that were significantly affected by the drug and by attention. Figure 3 shows the activity for the 3 different time periods of interest, for the 2 drugs used, separately for narrow- and broad-spiking cells and separated according to the attentional condition. Visual inspection shows that NMDAR blockade reduced the average neuronal activity for broad- and narrow-spiking cell for both attention conditions (Figure 3 top). The effect equally occurred when AMPARs were blocked (Figure 3 bottom). A very similar picture emerged, when all cells were included, irrespective of whether they were affected by the drug or attention.

**Figure 4:**
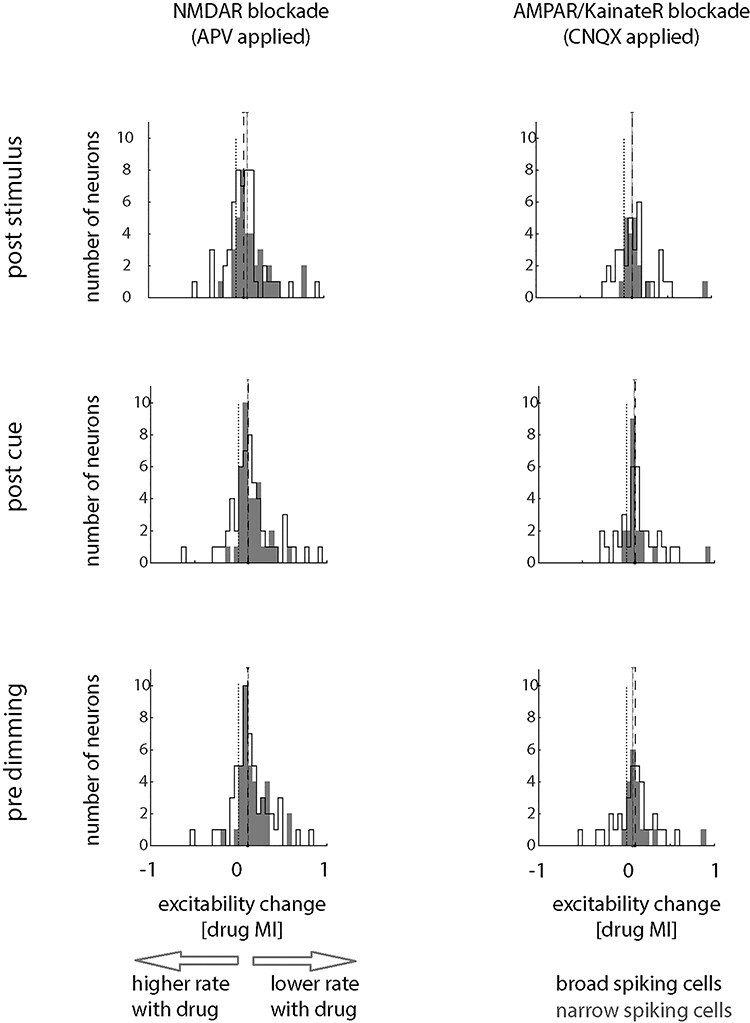
Effect of glutamatergic blockers on neuronal excitability, quantified through drug modulation indices (DrugMI, across attention conditions). Data for broad-spiking cells are shown by black outlined histograms, those for narrow-spiking cells by gray-filled histograms. The different drug conditions are shown separately for all 3 task periods (poststimulus, postcue onset, before the dimming aligned response period). Dashed lines within the histograms indicate medians for narrow- (gray) and broad-spiking cells (black), dotted line shows zero location.

To assess quantitatively how drug application affects neuronal excitability we calculated drug MIs. This was initially done for narrow- and for broad-spiking cells, and for all 3 relevant time periods (aligned to stimulus onset, to cue onset, and time of first dimming). We included cells significantly affected by drug application (2-factor ANOVA, main effect of drug or drug^*^attention interaction, see Table 1 for sample sizes of affected vs. nonaffected cells). This subselection was required, as we were interested in the effects of the drugs on neuronal activity, which cannot be assessed in cells where drugs did not have any effects. NMDAR and AMPAR blockade significantly reduced firing rates in both attention conditions (attend RF and attend away), in both cell types and all 3 analysis periods. This resulted in drug MIs significantly >0 for broad- and for narrow-spiking cells. Figure 4 shows distributions of MIs. The associated means, CIs, *P* and *t*-values, and effect sizes (Cohen’s *D*) are listed in Table 2. The effect of NMDAR blockade on the 2 cell types were not significantly different (Table 2 for details). Equally, the effect of AMPAR blockade on drug MIs did not differ significantly between cell types (Table 2 for details). The basic finding, namely that drug application did not differ between cell types, also held true when we analyzed the different cell clusters (Methods and Figure 1*C*) under NMDAR blockade. While drug application reduced firing rates in all cell clusters, this reduction did not differ between clusters (APV applied, *F*_4,86_ = 0.9, *P* = 0.4922, η^2^_p_ = 0.038). However, AMPAR blockade efficacy differed between different clusters (CNQX applied, *F*_4,47_ = 3.2, *P* = 0.020, η^2^_p_ = 0.214, mixed-model ANOVA). Post hoc comparison revealed that cluster N1 significantly differed from cluster B2 (*P* = 0.022, FDR corrected) and B2 differed from N2 (*P* = 0.022, FDR corrected).

**Table 2:** Effect of drug application on neural activity (quantified as modulation indices) during different task periods

**NMDAR blockade**					
**Narrow**					
	*t*_1,36_	*P*	Mean	CI	Cohen’s *D*
Precue	5.431	<0.001	0.177	0.111, 0.243	0.893
Postcue	6.490	<0.001	0.150	0.103, 0.197	1.067
Predim	6.254	<0.001	0.155	0.155, 0.206	1.028
**Broad**					
	*t*_1,53_	*P*	Mean	CI	Cohen’s *D*
Precue	3.162	0.003	0.093	0.034, 0.152	0.430
Postcue	3.995	<0.001	0.143	0.071, 0.214	0.554
Predim	4.264	<0.001	0.131	0.069, 0.193	0.580
**Narrow/broad difference**					
	*t*_1,89_	*P*	Mean (difference)	CI	Cohen’s *D*
Precue	1.883	0.063	−0.084	−0.005, 0.173	0.402
Postcue	0.150	0.881	−0.007	−0.087, 0.101	0.032
Predim	0.571	0.571	−0.024	−0.060, 0.109	0.122
**AMPA blockade**					
**Narrow**					
	*t*_1,18_	p	mean	CI	Cohen’s *D*
Precue	5.431	0.009	0.139	0.040, 0.239	0.675
Postcue	6.490	0.007	0.138	0.042, 0.234	0.695
Predim	6.254	0.007	0.136	0.043, 0.229	0.703
**Broad**					
	*t*_1,32_	*P*	Mean	CI	Cohen’s *D*
Precue	3.215	0.003	0.110	0.040, 0.180	0.560
Postcue	3.075	0.004	0.108	0.036, 0.179	0.535
Predim	2.055	0.048	0.078	0.001, 0.156	0.358
**Narrow/broad difference**					
	*t*_1,50_	*P*	Mean (difference)	CI	Cohen’s *D*
Precue	0.510	0.612	−0.029	−0.086, 0.145	0.147
Postcue	0.528	0.600	−0.030	−0.085, 0.146	0.152
Predim	0.951	0.346	−0.058	−0.064, 0.179	0.274

Note: Table displays *t*-statistics (along with df), significance, means of distributions and mean differences, CI and effect size (Cohen’s *D*) for the 2 drug regimens and for narrow- and broad-spiking cells.

### Drug Effects on Attentional Modulation of Neuronal Activity

From Figures 3 and 4 it appears that glutamatergic receptor manipulations affected the size of the attentional signal in some cells and for some conditions at the population level. To investigate this in detail, we quantified attentional modulation by calculating the AUROC and Cohen’s *D*′ for the attend RF versus attend away conditions in the 2 cell types. We took different approaches for cell inclusion. Initially, we included cells where drug application had a significant effect on neuronal activity, and where attention had a significant effect on neuronal activity, as we were interested in the drug effect (factor 1) on attentional modulation (factor 2) of firing rates, and thus, we reasoned both factors needed to be present. We will first report the results from this analysis, followed by reporting results from additional inclusion criteria.

AUROCs did not significantly depend on glutamate blockade (*F*_2,254_ = 1.2, *P* = 0.306, η^2^_p_ = 0.009 mixed model [MM-] ANOVA) or on cell type (*F*_1,254_ = 2.2, *P* = 0.143, η^2^_p_ = 0.008, MM-ANOVA). There was also no significant interaction between drug application and cell type (*F*_2,254_ = 0.5, *P* = 0.585, η^2^_p_ = 0.004, MM-ANOVA). Although the MM-ANOVA did not reveal any significant effect of drug application on attentional modulation quantified with AUROC, we nevertheless separately analyzed AUROC-based attentional modulation for the different drugs applied and for the 2 cell types to ensure no trending effects were missed. Blocking NMDARs did not reduce attentional modulation (quantified as AUROC) in broad- or in narrow-spiking cells (Table 3 for details). Blockade of AMPARs equally did not affect attentional modulation quantified by AUROCs (Table 3 for details).

**Table 3:** Effect of drug application on attentional AUROCs for different cell types/clusters

**NMDAR blockade**						
Cell type	df	*t*	*P*	Mean of differences	CI	*D*_z_
Broad	(1,48)	0.421	0.675	0.004	−0.015, 0.023	0.027
Narrow	(1,35)	1.208	0.235	0.013	−0.009, 0.035	0.096
N1(cluster)	(1,30)	1.174	0.249	0.013	−0.010, 0.036	0.211
B1(cluster)	(1,33)	0.945	0.352	0.010	−0.011, 0.031	0.162
B2(cluster)	(1,6)	-1.655	0.149	−0.050	−0.124, 0.024	-0.626
B3(cluster)	(1,2)	1.821	0.210	0.067	−0.092, 0.226	1.052
N2(cluster)	(1,9)	0.364	0.742	0.008	−0.041, 0.056	0.115
**AMPAR blockade**						
Broad	(1,26)	1.048	0.304	0.004	−0.016 0.050	0.105
Narrow	(1,17)	0.053	0.958	0001	−0.026 0.027	0.004
N1(cluster)	(1,17)	0.055	0.957	0.001	−0.027 0.029	0.013
B1(cluster)	(1,11)	1.596	0.139	0.035	−0.011 0.082	0.461
B2(cluster)	(1,2)	-0.948	0.443	−0.066	−0.366 0.234	-0.458
B3(cluster)	(1,5)	0.967	0.378	0.038	−0.063 0.138	0.395
N2(cluster)	(1,5)	0.092	0.930	0.002	−0.042 0.045	0.038

Note: Table displays *t*-statistics (along with df), significance, means of AUROC differences (AUROC control−AUROC drug), CI, and effect size (*D*_z_) for the 2 drugs investigated.

**Figure 5:**
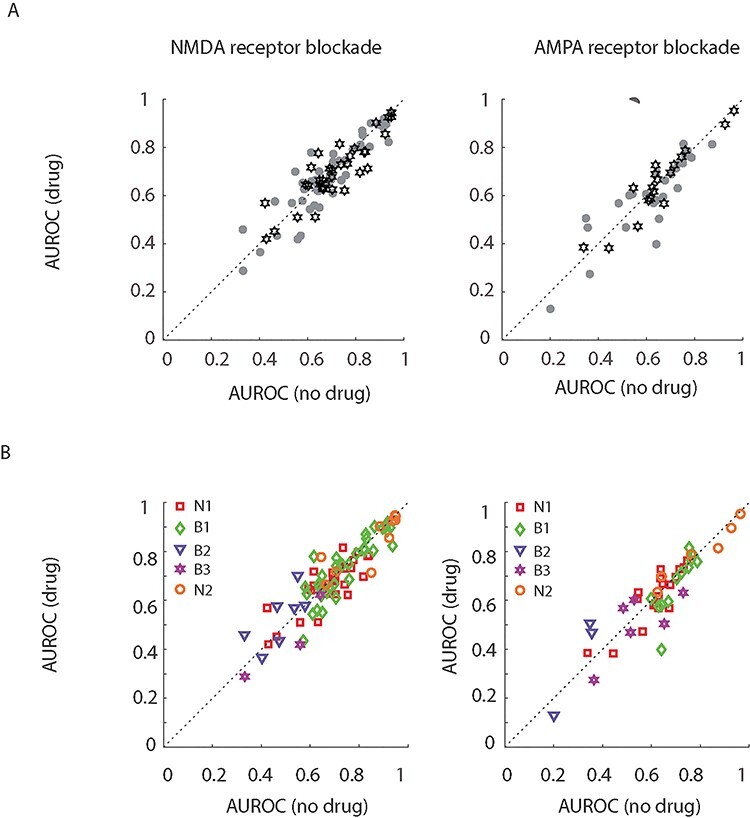
Attentional modulation quantified by calculating the AUROC when no drug (abscissa) was applied and when the drug of interest was applied (ordinate). (*A*) Black data points delineate AUROC values of narrow-spiking cells, gray data points those of broad-spiking cells. (*B*) Same as in (*A*), but for the 5 cell clusters identified by cluster analysis (B1–3, N1, 2).

In Figure 5A a minority of cells show AUROC values of <0.5 in the no drug condition. This implies that these neurons showed lower activity on attend RF trials than attend away trials, a phenomenon already described in previous publications (Thiele et al*.* 2016). These neurons showed activity reduction after cue onset, which was larger for attend RF than for attend away conditions, and we also find this in the current data set. It might be argued that for these cells the AUROC should be calculated as (1−AUROC). Doing so, slightly changed the reported overall significance level, as it resulted in significant drug effect (drug *F*_2,254_ = 3.4, *P* = 0.0365, η^2^_p_ = 0.026). Post hoc testing revealed that there were trending effects for narrow-spiking cells when NMDARs were blocked (*t*_1,35_: 1.917, *P* = 0.063, diff: 0.020, CI: −0.001, 0.041, *D*_z_: 0.162), and there were trending effects when AMPARs were blocked for broad-spiking cells (t_1,26_, *P* = 0.051, diff: 0.031, CI: −0.001, 0.062, *D*_z_: 0.285). No trending effects were found for NMDAR blockade in broad-spiking cells or AMPAR blockade in narrow-spiking cells.

Overall, similar results were obtained when we quantified attentional modulation using Cohen’s *D*. Here, we equally find that neither NMDAR nor AMPAR/KainateR blockade reduced attentional Cohen’s *D* in either broad-spiking cells (NMDAR blockade: *t*_1,48_ = −0.607, *P* = 0.547, diff: 0.020, CI: −0.047, 0.087, *D*_z_: 0.087; AMPAR blockade: *t*_1,26_ = 0.589, *P* = 0.555, diff: 0.026, CI: −0.064, 0.016, *D*_z_: 0.015, 2-sided *t*-test) or in narrow-spiking cells (NMDAR blockade: *t*_1,35_ = 0.935, *P* = 0.365; AMPAR blockade: *t*_1,17_ = −0.606, *P* = 0.552, diff: −0.030, CI: −0.134, 0.074, *D*_z_: −0.143, 2-sided *t*-test).

We next analyzed whether a different cell inclusion criterion altered our results. We therefore included all cells into the analysis, irrespective of whether they showed drug or attention effects individually. This selection did not change the results. AUROCs did not significantly depend on glutamate blockade (*F*_2,432_ = 2.8, *P* = 0.063, η^2^_p_ = 0.013, MM-ANOVA), but AUROC differed for the 2 cell types (*F*_1,432_ = 6.4, *P* = 0.012, η^2^_p_ = 0.015, MM-ANOVA). There was no significant interaction between drug application and cell type (*F*_2,432_ = 0.4, *P* = 0.681, η^2^_p_ = 0.002, MM-ANOVA). Although the MM-ANOVA did not reveal a significant effect of drug application on attentional modulation quantified with AUROC, it was trending. We thus separately analyzed AUROC-based attentional modulation for the different drugs applied and for the 2 cell types to ensure no trending effects were missed. Blocking NMDARs did not reduce attentional modulation (quantified as AUROC) in broad- or in narrow-spiking cells (broad: *t*_1,81_ = −0.572, *P* = 0.569, diff: 0.004, CI: −0.016, 0.009, *D*′: 0.02; narrow: *t*_1,52_ = 0.576, *P* = 0.576, diff: 0.005, CI: −0.012, 0.021, *D*′: 0.035, respectively, 2-sided *t*-test). Blockade of AMPARs equally did not affect attentional modulation quantified by AUROCs, (narrow [*t*_1,32_ = 1.347, *P* = 0.187,, diff: 0.012, CI: −0.006, 0.030, *D*′: 0.084, 2-sided *t*-test; broad: *t*_1,50_ = 1.412, *P* = 0.161, diff: 0.004, CI: −0.006, 0.030, *D*′: 0.084, 2-sided t-test). This shows that even if all cells are included into the analysis, there was no effect of either drug on attentional modulation. Doing further subselections, based on cells affected by drug irrespective of attention effects, or affected by attention irrespective of drug effects, yielded the same qualitative outcomes.

### Cell-Type by Cluster Analysis

We next asked the question whether subdividing cells further based on the cluster-analysis might reveal significant effects of iGluR blockade on attentional modulation for some of the clusters. For cells significantly affected by attention and drug application, we found no main effect of drug on attentional modulation (*F*_2,245_: 0.5, *P* = 0.601, η^2^_p_ = 0.004), but we found a significant drug ^*^ cell-type interactions (*F*_8,245_: 2.0, *P* = 0.041, η^2^_p_ = 0.063). However, post hoc analysis did not reveal any significant changes to attentional AUROCs in any of the cell clusters (Fig. 5*B*, Table 3 for associated *t*-, *P* values, differences, and effect sizes). As done above for the narrow–broad-spiking divide, we performed this analysis on our entire data set of cells, that is, irrespective of whether individual cells were significantly affected by attention or drug. For the full data sample, we found that AUROC significantly depended on drug application: (*F*_2,423_ = 3.3, *P* = 0.036, η^2^_p_ = 0.016, MM-ANOVA), but there was no significant interaction between cell-type^*^drug (*F*_8,423_ = 1.2, *P* = 0.285, η^2^_p_ = 0.023, MM-ANOVA). Post hoc analysis showed that this was due to a change in attentional AUROCs in the B2 cluster upon NMDAR blockade, that is, in the cluster where attention decreased neuronal firing rates (*t*_1,18_ = −2.318, *P*(FDR adjusted) = 0.032, diff: −0.032, CI −0.061, −0.003, D′: −0.532). Thus, if there was an effect of local iGluR blockade on attentional modulation, then it was restricted to NMDAR blockade in cells where attention reduces firing rates for the attend RF condition. Note that increases of AUROCs in these cells do not imply that attentional modulation was increased, but rather that it was decreased (i.e., AUROCs getting closer to 0.5). However, the effects were only present when analyzing all cells (irrespective of attention or drug effects at the single-cell level) and effect sizes were small.

### Subdividing Cells According to Response Characteristics

We next analyzed whether an analysis of drug effects according to response classification would yield different effects. As described in methods we separated cells into “visual” (*n* = 57; visual response transient dominates the characteristics, Methods for details), “visuo-attention” (*n* = 70; visual response transient is present, but activity before first dimming in attend RF conditions is almost as large or larger than visual transient response), “attention” (*n* = 19; visual response mostly absent, whereas activity before first dimming in attend RF conditions is at least 20% larger than baseline activity), and “other” (*n* = 71) response types. We analyzed for each response type whether attentional modulation (as measured by AUROC) was affected by NMDAR or AMPAR blockade. We did this 1) irrespective of whether cells were affected by attention or drug application, 2) for cells significantly affected by attention, irrespective of drug effects, 3) for cells significantly affected by drug application, irrespective of attention effects, and 4) for cells significantly affected by attention and by drug application.

Neither approach showed significant effects of drug on attentional modulation in any of the cells with different response types (approach 1: cell-type *F*_3,434_ = 2.2, *P* = 0.083, η^2^_p_ = 0.015; drug *F*_2,434_ = 0.6, *P* = 0.571, η^2^_p_ = 0.003; cell-type^*^drug interaction *F*_6,434_ = 1.4, *P* = 0.206, η^2^_p_ = 0.019; approach 2: cell-type *F*_3,384_ = 1.9, *P* = 0.125, η^2^_p_ = 0.015; drug *F*_2,384_ = 0.2, *P* = 0.788, η^2^_p_ = 0.001; cell-type^*^drug interaction *F*_6,384_ = 1.3, *P* = 0.238, η^2^_p_ = 0.020; approach 3: cell-type *F*_3,278_ = 2.3, *P* = 0.074, η^2^_p_ = 0.025; drug *F*_2,278_ = 0.2, *P* = 0.828, η^2^_p_ = 0.001; cell-type^*^drug interaction *F*_6,278_ = 0.9, *P* = 0.503, η^2^_p_ = 0.019; approach 4: cell-type *F*_3,250_ = 1.9, *P* = 0.134, η^2^_p_ = 0.022; drug *F*_2,250_ = 0.1, *P* = 0.911, η^2^_p_ = 0.001; cell-type^*^drug interaction *F*_6,250_ = 1.0, *P* = 0.456, η^2^_p_ = 0.022, MM-ANOVA). Performing individual paired *t*-tests on the data (separate for response type, drug and subselection) equally yielded no significant effects on attentional AUROCs, despite that fact that it entailed multiple tests, with accordingly increased changes of false-positives (and if they had been significant would need correction for multiple comparison). Thus, the absence of local iGluR blockade on attentional modulation in FEF neurons was not due to the fact that we ignored cell type response properties.

### Firing Rate Variability as a Function of Attention and Drug Application

**Figure 6:**
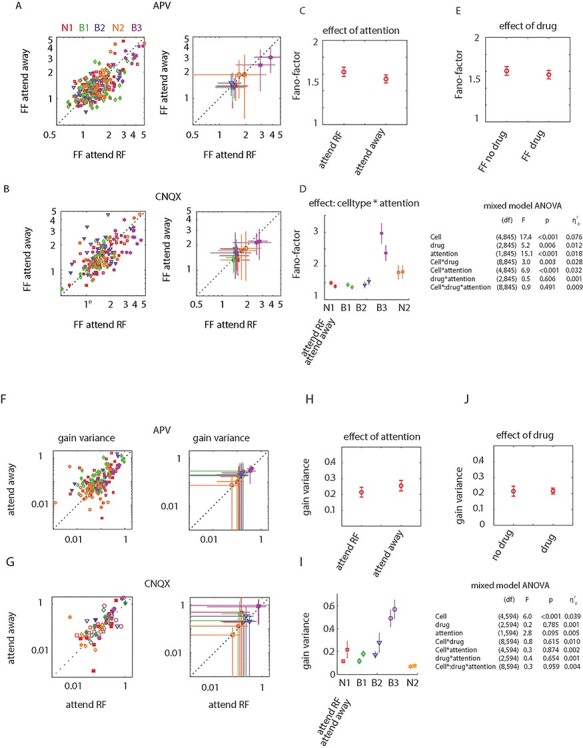
Neuronal variability quantified by FFs and gain variance for the different drug and attention conditions for the 5 different cell clusters. (*A*) Individual FF data points (right) when NMDARs were blocked. Open symbols—no drug applied, filled symbols—drug applied. Mean FFs and standard deviation for the 5 different clusters are shown to the right. (*B*) Same as (*A*) but for the cells without and with AMPA receptors blocked. (*C*) mean ± SEM effect of attention on FFs across all cells. (*D*) Mean ± SEM effect of attention on FFs for the 5 different cell clusters. Table to the right of (*D*) shows significance of effects along with effect size (MM-ANOVA). (*E*) Comparison of FF mean ± SEM without and with drug applied. (*F*–*J*) same as (*A*–*E*), but for gain variance calculations.

In our previous study of glutamatergic modulation of attentional signals in macaque V1, we found that NMDARs contributed to attention-induced reduction of firing rate variability, whereas it did not affect attention-induced alterations of firing rate itself (Herrero et al*.* 2013). To investigate whether similar results hold for the FEF, we quantified rate variability by calculating FF = variance of rate/mean rate as well as by calculating gain variance (Goris et al*.* 2014; Thiele et al*.* 2016). For this analysis, we do not specifically select cells based on significant attention or effects on firing rate, as firing rate variance could be affected independently from firing rate itself. Thus, the entire cell sample was analyzed. Using the cell-type assignment along the broad/narrow divide, we found that FFs significantly differed between attention conditions, whereby FFs were slightly, but significantly smaller in attend away conditions (*F*_1,863_ = 9.5, *P* < 0.003, η^2^_p_ = 0.011, MM-ANOVA). Neither drug (*F*_2,863_: 1.4, *P* = 0.236, η^2^_p_ = 0.003, MM-ANOVA), nor cell-type (broad/narrow spiking) had a significant effect on FF (*F*_1,863_: 0.1, *P* = 0.793, η^2^_p_ < 0.001), nor were there any significant interactions (largest *F*_2,863_:0.8, smallest *P* = 0.367, largest η^2^_p_ = 0.001, MM-ANOVA). More nuanced results were obtained using the cell-type assignment based on the cluster approach. Figure 6*A* shows FFs for the 5 cell clusters for attend RF versus attend away trials for no drug (open symbols) and for conditions when NMDA receptors were blocked (filled symbols). The equivalent data for no drug and AMPA receptor blockade are shown in Figure 6*B*. Figure 6*C*–*E* shows mean and standard error of mean (SEM) values for factors that significantly affected FFs (the MM-ANOVA table including effect sizes is given as an inset below Fig. 6*E*). Attention to the RF resulted in slightly increased FFs. However, this effect depended on which cluster was analyzed. Attention to the RF resulted in increased FFs in clusters N1, B1, and B3. However, the opposite pattern was found in clusters B2 and N2, where attention resulted in slightly reduced FFs. Overall FFs differed between cell types, but the latter was expected, as it was a clustering variable.

The fact that FFs were reduced by drug application and overall increased by attention is only surprising at first glance. As argued previously, FFs are affected by overall firing rate, due to the nonlinear, expansive relationship between mean rate and rate variance (Thiele et al*.* 2016). Thus, with increasing firing rates, FFs will become larger, as would be the case with attend RF conditions. Since drug application reduced firing rates, FFs would decrease as seen in Figure 6*E*. Analysis of gain variance (Goris et al*.* 2014; Thiele et al*.* 2016) is designed to eliminate the above-mentioned problem. Gain variance quantifies neuronal variability that exceeds the variability induced by the spike renewal process (Poisson variability) and thus quantifies fluctuations in neural excitability. We calculated gain variance as a function of attention and drug application for each cell recorded (Fig. 6*F*–*J*).

Gain variance trended to be larger for attend away conditions than attend RF conditions (*F*_1,594_ = 2.8, *P* = 0.095); it differed between cell types (*F*_4,594_ = 6.0, *P* < 0.001, MM-ANOVA, Fig. 6*F*–*H*). Gain variance did not depend on drug application (*F*_2,594_ = 0.2, *P* = 0.785), and there were no interactions between any of the other factors. The entire MM-ANOVA table, including effect sizes, is given as an inset below Figure 6*J*. Note that gain variance could not be calculated for cells where rate variance was smaller than rate mean (Goris et al*.* 2014); hence the number of cells contributing was smaller (*n* = 157).

### NMDAR Blockade and Its Effect on Attention Modulation in a 2 Stage Neuronal Network Model

**Figure 7:**
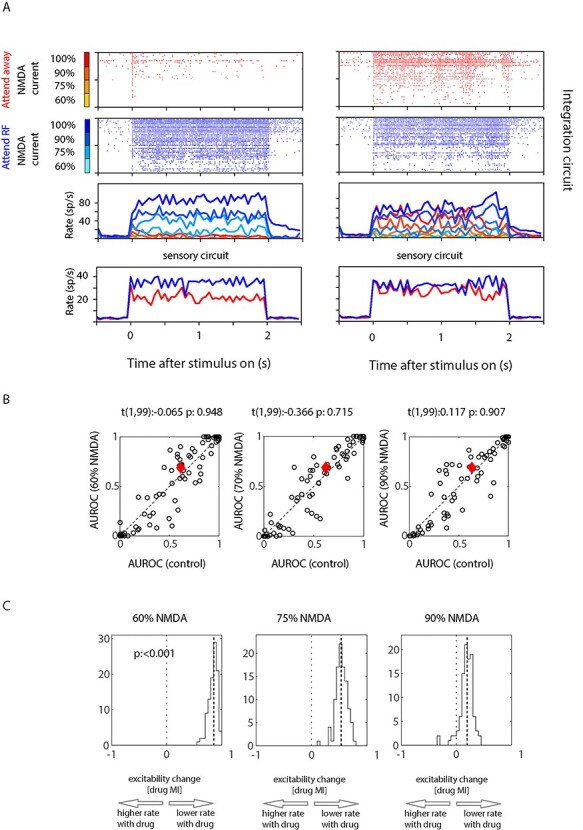
Attentional modulation in a 2-stage network model. (*A*) Two different instantiations of attentional modulation in a 2-stage network model. Left raster plots and histograms show an example where attentional modulation was large (AUROC > 0.99). Right raster plots and histograms where attentional modulation was more modest (AUROC ~ 0.78). The upper histograms show the mean activity in the integration circuit. Blue colors show activity in the attend RF circuit, red colors the activity in the attend away circuit. Different color shadings indicate whether NMDA currents were unaffected (100% NMDA) or whether they were reduced (percentages indicate the amount of NMDA currents still available). The raster plots show activities of single cells from the circuit (from bottom to top 10 successive cells show the same amount of NMDA current available, i.e., NMDA drive increases every 10 cells). (*B*) Attentional modulation quantified as AUROC for 100 network runs (black dots) calculated between activity from 10 cells with 100% NMDA current drive versus 10 cells with reduced NMDA current drive (NMDA drive as percentage to maximum). The red symbol in the center indicates median AUROC for the 2 contrasted conditions. (*C*) Rate modulation histograms with reduced NMDA current drive, quantified as attentional modulation index (drug MI). Median drug MI is indicated by the thick dashed lines.

We were wondering how glutamatergic activity can be dissociable from local modulation by attention, and how our results are compatible with a role of glutamatergic (NMDA) receptors in persistent activity. We believe that the effect seen in our study is a consequence of the fact that individual cells are embedded in large-scale populations with variable distance relationships. The excitatory drive any cell receives is temporally very heterogeneous (due to the large number of inputs). Thus, if excitatory drive was just mediated by AMPA currents, the activity can be persistent, even when NMDARs are blocked at the level of a single cell. Given our local iontophoresis, we affected only a limited number of cells, which most likely constitute a small fraction of the overall network. The majority of cells representing a specific attention location still would have intact NMDA currents and can develop the attentional modulation (which exhibits a soft form of winner take all behavior, Figure 3). We reasoned that these unaffected cells continue to drive the cells (by means of temporally dispersed AMPA currents) in which NMDARs were blocked. This drive would be strong during attend RF conditions, and weaker during attend away conditions. To test this proposal (for proof of concept, not for identification of specific detail), we modified an existing 2-stage neural network model (Wimmer et al*.* 2015) (model code is available at https://github.com/alex2thiele/attention-decision-model/tree/main, some details of the model are provided in Methods). We ran the model 100 times (random seed for the key parameters) and determined the attentional modulation (AUROC) and the drug rate modulation (Drug MI) in non-blocked cells and in NMDAR-blocked cells. The results for 2 selected runs are shown in Figure 7*A*. The attentional modulation for the unaffected versus differently NMDAR affected cells is shown in Figure 7*B*. The attentional AUROCs did not differ for cells affected versus unaffected for any of the NMDA reductions (paired *t*-test, *t*-statistics, and values are given in as insets in the figure). The Drug MI rate modulation is shown in Figure 7*C*. Even a 10% reduction on NMDA current in model cells resulted in stronger firing rate modulations than what we recorded in our neural data. Thus, the model replicates most of the intuition outlined above, namely that small numbers of neurons embedded in a larger population can retain their attentional modulation when NMDA receptors are blocked, as the larger population still has access to NMDA currents and continue to drive cells with blocked NMDA currents through temporally dispersed AMPA currents.

### Drug Effects on Behavioral Performance

Given the very local drug application, we did not necessarily expect drug application to alter performance in terms of either reaction times (RT), proportion of correct decisions, or fixational eye movements. This was indeed the case when NMDARs were blocked for RTs (ANOVA: main effect of drug: *F*_1,95 888_ = 0.32, *P* = 0.568, η^2^_p_ < 0.001; main effect of attention: *F*_1,95 888_ = 0.03, *P* = 0.9969, η^2^_p_ < 0.001, interaction: (*F*_2,95 888_ = 0.11, *P* = 0.744, η^2^_p_ < 0.001) and for the proportion of correct decisions (χ^2^ = 0.283, *P* = 0.601). However, when AMPARs were blocked RTs were increased (ANOVA: main effect of drug: *F*_1,60 189_ = 4.05, *P* = 0.044, η^2^_p_ < 0.001, mean RT difference [drug–no drug]: 0.761 ms, CI −1.154, −0.023, D′: −0.016; main effect of attention: *F*_1,60 189_ = 0.04, *P* = 0.9944, η^2^_p_ < 0.001; interaction: *F*_2,60 189_ = 0.1, *P* = 0.747, η^2^_p_ < 0.001). Note that despite the significance the amount of variance explained on RTs by drug application was less than 0.1%.

Fixational eye movements (microsaccade amplitude, velocity, direction, frequency, or mean *x*-, *y*-position) were not affected by AMPAR blockade (all *P* > 0.1, all η^2^_p_ < 0.001), However, we did observe effects of NMDAR blockade on microsaccade amplitude (*F*_1,161 446_ = 6.65, *P* = 0.009, η^2^_p_ < 0.0001, D′: −0.013, mean difference: −0.004, CI: −0.007 −0.001), and velocity (*F*_1,161 446_ = 8.59, *P* = 0.003, η^2^_p_ < 0.0001, *D*′: −0.014, mean difference: −0.361, CI: −0.59, −0.12), but effect sizes were overall very small. No effects were found by NMDAR blockade on microsaccade direction, frequency, or mean eye position (all *P* > 0.2, η^2^_p_ < 0.0001).

The frequency of correct decisions was not affected by drug application (all *P* > 0.1, all η^2^_p_ < 0.001).

## Discussion

Glutamatergic receptor blockade reduced excitability of broad- and narrow-spiking cells, of the 5 different cell groups identified by cluster analysis and of the specific response type a cell exhibited. These effects occurred irrespective of whether AMPA or NMDA receptors were blocked. Attentional modulation of firing rates was unaffected by local AMPA or NMDA receptor blockade in either broad- and narrow-spiking cells, and it was unaffected in cell groups identified by cluster analysis. The absence of an effect on attentional modulation can be explained by a network model where cells are embedded in large networks. Here inputs from the wider network (using different receptors) compensate for effects of local specific receptor blockade. Blockade of either receptor resulted in reduced firing rate variability, when assessed by FFs, not when assessed by gain variability. These data demonstrate that drug-induced reductions in firing rate does not automatically translate into reduction of attentional signals and that iGluRs are not themselves responsible for inducing attentional signals inFEF.

### Glutamatergic Contribution to Neuronal Excitability and Neuronal Firing Rate Variability

Glutamate is the main excitatory neurotransmitter in the brain (Hollmann and Heinemann 1994). It is thus no surprise that blockade of AMPA and NMDA receptors reduced neuronal excitability and neuronal firing rates in our study. This is in line with previous studies that reported reduction in firing rates when AMPA or NMDA receptors were blocked locally (Self et al*.* 2012; Herrero et al*.* 2013; Wang et al*.* 2013; Yang et al. 2018; van Vugt et al*.* 2020). However, systemic application of NMDAR antagonists (e.g., Ketamine) increased overall activity in DLPFC (Skoblenick and Everling 2012; Wang et al*.* 2013). The discrepancy between local and systemic application of the NMDAR antagonist could be explained by large scale network effects, where a change induced in remote locations/areas, results in altered input (e.g., feed-forward of feed-backward inhibition), with concomitant changes in firing rates at the recording location.

The contribution of AMPA and NMDA receptors to different aspects of neuronal activity has been reported for DLPFC and area V1. In DLPFC, an area close to FEF, NMDARs are more strongly involved in supporting working memory-related delay activity than AMPA receptors (Wang et al*.* 2013; but see van Vugt et al*.* 2020), whereas both receptors contribute to stimulus-driven activity. In primary visual cortex of the macaque, somewhat conflicting results have been reported. While the effect of AMPA receptor blockade on stimulus-driven activity in V1 cells is undisputed (Self et al*.* 2012; Herrero et al*.* 2013; Yang et al*.* 2018), the effect of NMDA receptor blockade was either small (when general NMDA antagonists were used; Self et al*.* 2012; Yang et al*.* 2018), resulted in increased activity when NR2B subunit NMDA receptors were blocked (Self et al*.* 2012), or caused response reductions similar to those seen when AMPA receptors were blocked (Herrero et al*.* 2013). The latter result is reminiscent of effects seen in cat V1 (Fox et al. 1990).

NMDA and AMPA receptors blockade had similar effects on cell excitability, irrespective of the cell types analyzed (narrow-/broad-spiking cells or different identified cell clusters). This differs from the effects of cholinergic receptor blockade in FEF. In relation to cholinergic receptor blockade, narrow-spiking cells were more strongly affected by muscarinic receptor blockade than broad-spiking cells. The absence of such differences upon IGluR blockade suggests that AMPA and NMDA receptors have similar expression levels on these cells types in FEF and that both receptors are equally involved in excitability. The similarity of NMDAR and AMPAR blockade on cell excitability in FEF contrasts with effects described for DLPFC (Wang et al*.* 2013). In DLPFC, NMDA-driven currents play a more importantrole.

Firing rate variability differed between different cell clusters, and attention differently affected the rate variability. Overall, attention increased rate variability when quantified using the FF. While this may be surprising (given the effects of attention on rate variability in sensory areas Mitchell et al*.* 2007; Niebergall et al. 2011; Herrero et al*.* 2013; Thiele et al*.* 2016), the effects in frontal cortex differ (Chang et al*.* 2012; Purcell et al. 2012; Thiele et al*.* 2016). Whether these differences are solely a consequence of the nonlinear relationship between firing rate and rate variance (Thiele et al*.* 2016), or whether differences in network structure and function contribute as well, is currently unknown. The effects of attention on rate variability were most pronounced in a subset of broad-spiking cells (cluster B3). These cells had comparatively large rate variability but otherwise were not unique in relation to the other cluster parameters (Fig. 1*C*). When rate variability was assessed using gain variance (Goris et al*.* 2014), we found a trend toward reduced gain variance with attention, which appeared smallest in cluster N2 (the cluster with comparatively large firing rate and strong attentional modulation).

While it is tempting to map these clusters onto physiological cell types, we believe this is not possible with our methodology. Narrow-spiking cells are often argued to be GABAergic (fast-spiking) interneurons, whereas broad-spiking cells have been argued to be predominantly pyramidal cells (Mitchell et al*.* 2007; Hussar and Pasternak 2012; Jacob et al. 2013; Ott et al. 2014; Jacob et al. 2016). This mapping is an oversimplification even in rodents, where narrow-spiking cells can comprise a variety of different cell types (Tremblay et al. 2016; Yavorska and Wehr 2016), but it could be plain wrong in primates (Vigneswaran et al*.* 2011; Thiele et al*.* 2016; Soares et al*.* 2017; Dasilva et al*.* 2019). Thus, assigning cell type labels to clusters requires independent verification, which was not available in this study.

### Glutamatergic Contribution to Attentional Modulation

While we found individual cell examples where AMPAR or NMDAR blockade resulted in significantly changed attentional modulation (Figure 2), we did not find any systematic changes at the level of the recorded cell population, irrespective whether assessed across all cell, across the narrow–broad-spiking divide, across the cluster-based divide, or according to response type differences. This clearly sets the data apart from our recent finding that cholinergic blockade reduces attentional signals in FEF (Dasilva et al*.* 2019). Muscarinic receptors contributed to attentional signals in broad- and narrow-spiking cells (and across almost all cell clusters that were analyzed), whereas nicotinic receptor blockade caused attentional modulation reduction only in a specific narrow-spiking cell cluster. The difference between cholinergic and glutamatergic effects on attentional rate modulation results in an important conclusion, namely that similar reductions in absolute firing rate do not necessarily cause reductions of attentional modulation. By extension, attentional signals in individual cells are not solely the result of excitatory drive through specific iGluRs (and associated neuronal depolarization levels). We were nevertheless surprised that NMDAR blockade did not cause attentional rate modulation in FEF when applied locally, given its contribution to spatial working memory signals in neighboring DLPFC (Wang et al*.* 2013; Yang et al. 2013). Our working hypothesis was that spatial top-down attention is conceptually similar to spatial working memory, as a location has to be monitored/held in memory for extended periods of time in both conditions. One important difference of course is the constant presence of a stimulus in the receptive/memory field in our task condition, which is not present in a working memory task. It is possible that the constant excitatory drive induced by the stimulus is sufficient to allow the network to maintain/produce the attentional signal even when the intrinsic excitation is reduced by NMDAR blockade. Alternatively, the embedding into a large network of cells, resulting in temporally dispersed AMPA receptor activation, might enable individual cells to replicate the behavior of the larger population (i.e., the attentional modulation), as simulated in our 2-stage network model. However, even if that was the case, it still sets the iGluR system apart from the cholinergic system where identical task and stimulus conditions resulted in different outcomes. Why this is the case is currently unknown. In DLPFC release of the Mg^++^ block of NMDARs is mediated through α7 nicotinic receptors (Wang et al*.* 2013), which enables the network to generate persistent delay activity in the absence of sensory stimulation. This is different to the mechanisms in sensory areas where AMPAR-dependent depolarization (upon sensory stimulation) releases the Mg^++^ block of NMDARs (Yang et al*.* 2018). It could be that blockade of α7 nicotinic receptors has a stronger effect on the neuronal ability to integrate signals over time, than NMDARs have by themselves (in DLPFC). However, it is likely that blockade of, for example, M1 receptors, and associated unblocking of the M-current (thereby making cells more leaky), has a strong effect on temporal integration and associated development of attentional signals. This could explain the difference between cholinergic and glutamatergic effects seen (Dasilva et al*.* 2019). This possibility could be explored by a more detailed network model, but such a model is beyond the scope of the current paper.

Our current data set differs from the glutamatergic effects in V1, as local glutamatergic blockade in FEF had no effect on attentional modulation of rate variability, whereas glutamatergic NMDAR blockade reduced the effects of attention on rate variability in V1 (Herrero et al*.* 2013). This cannot simply be the consequence of limited effects of attention on rate variance, as the effect of attention on FFs was significant, even if unexpected. We believe that our results argue for an important role of glutamatergic signaling to maintain overall excitability in FEF, whereby large-scale network interactions are required to generate persistent delay activity and attentional signals.

## Funding

Funded by the Wellcome Trust (093104); Biotechnology and Biological Sciences Research Council UK (BB/F021399/1); Medical Research Council UK (MR/P013031/1).

## Notes

We thank the Comparative Biology Centre staff at Newcastle University for their excellent technical support and Prof. Stuetzel for providing histology laboratory space. We also thank Klaus Wimmer for advice on with setting up the network model. *Conflict of Interest*: None declared.

## Author Contribution

Study conception and planning: A.T., data acquisition: M.D., C.B., histology and recording site verification: C.D., data analysis: A.T., manuscript writing: A.T.,M.D.
